# Dietary Patterns Are Associated with Predicted 10-Year Risk of Cardiovascular Disease Among Ghanaian Populations: the Research on Obesity and Diabetes in African Migrants (RODAM) Study

**DOI:** 10.1093/jn/nxz002

**Published:** 2019-04-24

**Authors:** Daniel Boateng, Cecilia Galbete, Mary Nicolaou, Karlijn Meeks, Erik Beune, Liam Smeeth, Hibbah Araba Osei-Kwasi, Silver Bahendeka, Peter Agyei-Baffour, Frank P Mockenhaupt, Joachim Spranger, Diederick E Grobbee, Matthias B Schulze, Karien Stronks, Charles Agyemang, Ina Danquah, Kerstin Klipstein-Grobusch

**Affiliations:** 1 Julius Global Health, Julius Center for Health Sciences and Primary Care, University Medical Center Utrecht, Utrecht University, Utrecht, The Netherlands; 2 School of Public Health, Kwame Nkrumah University of Science and Technology, Kumasi, Ghana; 3 Department of Molecular Epidemiology, German Institute of Human Nutrition Potsdam-Rehbruecke, Nuthetal, Germany; 4 Department of Public Health, Amsterdam UMC, University of Amsterdam, Amsterdam Public Health Research Institute, Amsterdam, The Netherlands; 5 Department of Non-communicable Disease Epidemiology, London School of Hygiene and Tropical Medicine, London, United Kingdom; 6 Public Health Section, School of Health and Related Research–ScHARR, University of Sheffield, Sheffield, United Kingdom; 7 Department of Clinical Sciences and Nutrition, University of Chester, Chester, United Kingdom; 8 MKPGMS – Uganda Martyrs University, Kampala, Uganda; 9 Institute of Tropical Medicine and International Health, Charité – University Medicine Berlin, Berlin, Germany; 10 Charité Center for Cardiovascular Research (CCR), Berlin, Germany; 11 Division of Epidemiology & Biostatistics, School of Public Health, Faculty of Health Sciences, University of the Witwatersrand, Johannesburg, South Africa

**Keywords:** dietary patterns, cardiovascular disease risk, pooled cohort equation, Ghana, migrants, sub-Saharan Africa, RODAM study

## Abstract

**Background:**

Sub-Saharan African populations are disproportionately affected by cardiovascular disease (CVD). Although diet is an important lifestyle factor associated with CVD, evidence on the relation between dietary patterns (DPs) and CVD risk among sub-Saharan African populations is limited.

**Objective:**

We assessed the associations of DPs with estimated 10-y atherosclerotic cardiovascular disease (ASCVD) risk in Ghanaian adults in Ghana and Europe.

**Methods:**

Three DPs (‘mixed’; ‘rice, pasta, meat, and fish’; and ‘roots, tubers, and plantain’) were derived by principal component analysis (PCA) based on intake frequencies obtained by a self-administered Food Propensity Questionnaire in the multi-center, cross-sectional RODAM (Research on Obesity and Diabetes among African Migrants) study. The 10-y ASCVD risk was estimated using the Pooled Cohort Equations (PCE) for 2976 subjects, aged 40–70 y; a risk score ≥7.5% was defined as ‘elevated’ ASCVD risk. The associations of DPs with 10-y ASCVD risk were determined using Poisson regression with robust variance.

**Results:**

Stronger adherence to a ‘mixed’ DP was associated with a lower predicted 10-y ASCVD in urban and rural Ghana and a higher 10-y ASCVD in Europe. The observed associations were attenuated after adjustment for possible confounders with the exception of urban Ghana (prevalence ratio [PR] for Quintile 5 compared with 1: 0.70; 95% CI: 0.53, 0.93, *P*-trend = 0.013). The ‘rice, pasta, meat, and fish’ DP was inversely associated with 10-y ASCVD across all study sites, with the adjusted effect being significant only in urban Ghana. A ‘roots, tubers, and plantain’ DP was directly associated with increased 10-y ASCVD risk.

**Conclusions:**

Adherence to ‘mixed’ and ‘rice, pasta, meat, and fish’ DPs appears to reduce predicted 10-y ASCVD risk in adults in urban Ghana. Further investigations are needed to understand the underlying contextual-level mechanisms that influence dietary habits and to support context-specific dietary recommendations for CVD prevention among sub-Saharan African populations.

## Introduction

The management of cardiovascular disease (CVD) preventive measures has been improving steadily over the last decade (1). Deaths from CVD have been dramatically reduced in many high-income countries (1), owing to the promotion of healthier lifestyles and providing equitable healthcare by instituting appropriate government policies (2). In contrast, it is an increasing developmental issue in low- and middle-income countries (LMIC) (3), with over 80% of CVD deaths occurring in these countries (4). This has been linked to progressive urbanization, the globalization of unhealthy lifestyles, and lack of equitable healthcare (5, 6). Furthermore, migrants originating from LMIC are disproportionately affected by CVD (7). Recent findings from the multi-center Research on Obesity and Diabetes in African Migrants (RODAM) study among Ghanaians also show an increased prevalence of diabetes and obesity (8) and estimated CVD risk (9) among European migrant populations compared with their home counterparts. The causes of these differences in CVD risk between migrant populations and their host and native home populations are not fully understood.

Diet is an important lifestyle factor associated with CVD (10, 11). Individual nutrients and foods, however, cannot be considered in isolation due to the complex interactions among nutrients and because the effect of a single nutrient on multifactorial health outcomes such as noncommunicable diseases may be undetectable (12, 13). Dietary patterns (DPs) are therefore required to fully understand the overall influence of diet on CVD risk (14). DPs characterized by sweets, rice, meat, fruits, and vegetables have been associated with a decreased risk of type 2 diabetes (T2D) among Ghanaian migrant and home populations (15, 16), whereas a DP characterized by fruits, vegetables, tubers, and legumes has been linked with a reduced risk of hypertension in Cameroon (17).

Globalization and economic development in LMIC have resulted in a change in diet towards a more ‘Western diet’, defined by the high intake of added sugars, fats, refined carbohydrates, and animal-source foods (18). Migration to high-income countries also comes with potential changes in diet that are due to changes in the physical environment and adoption of dietary behavior of the host country (19). A recent study among Ghanaian migrants in the United Kingdom (UK) found the adoption of key features of UK food practices (20). An enculturation of indigenous foods (21) and improved dietary diversity upon migration has also been observed among Ghanaian migrants in Europe (22). These dietary changes have been associated with a risk of CVD as well as obesity and T2D (23, 24).

Data on the dietary habits and estimated risk of CVD among sub-Saharan African migrant populations and their home country counterparts remain limited. Recently, DPs among adult Ghanaians residing in Ghana and Europe were described within the RODAM study (21) and the relation between DPs and T2D has been established (15, 22). However, the role of DPs in relation to estimated CVD risk among this population has not been elucidated. Assessing the combined effect of risk factors has been reiterated as a more effective strategy, compared with individual risk factors, for delivering CVD prevention interventions (25). Using an established risk algorithm (26), estimated CVD risk will help gain insight into the association of dietary habits and the possibility of a cardiovascular event within a specified period of time. The aim of this study was to assess the association between DPs and estimated 10-y CVD risk among Ghanaian populations living in Europe and their compatriots in Ghana.

## Methods

### Study design and study population

Details of the multi-center RODAM study including the recruitment and sample size estimations have previously been published (27). In brief, this multi-center cross-sectional study was conducted among Ghanaian adults in rural Ghana, urban Ghana, and Europe (Amsterdam, London, and Berlin) between July 2012 and September 2015 (*n* = 6385). For recruitment in Ghana, census data of 2010 were used to select rural and urban participants in the Ashanti Region. In Amsterdam, the Municipal Health Register was used to randomly select Ghanaian migrants who were invited by postal mail and home visits. In London and Berlin, Ghanaian organizations, church communities, and social unions served as the sampling frame for recruitment. The response rates were 76% in rural Ghana and 74% in urban Ghana. In Amsterdam, 67% replied by response card or after a home visit. Of these, 53% agreed and participated in the study. In London, of those individuals who were invited based on their registration in Ghanaian organizations, 75% agreed and participated in the study. In Berlin, this figure was 68%. The RODAM study was conducted according to the guidelines laid down in the Declaration of Helsinki. All procedures involving human subjects were reviewed and approved by the respective ethics committees in Ghana, the Netherlands, the UK, and Germany. Written informed consent was obtained from all participants.

**FIGURE 1:**
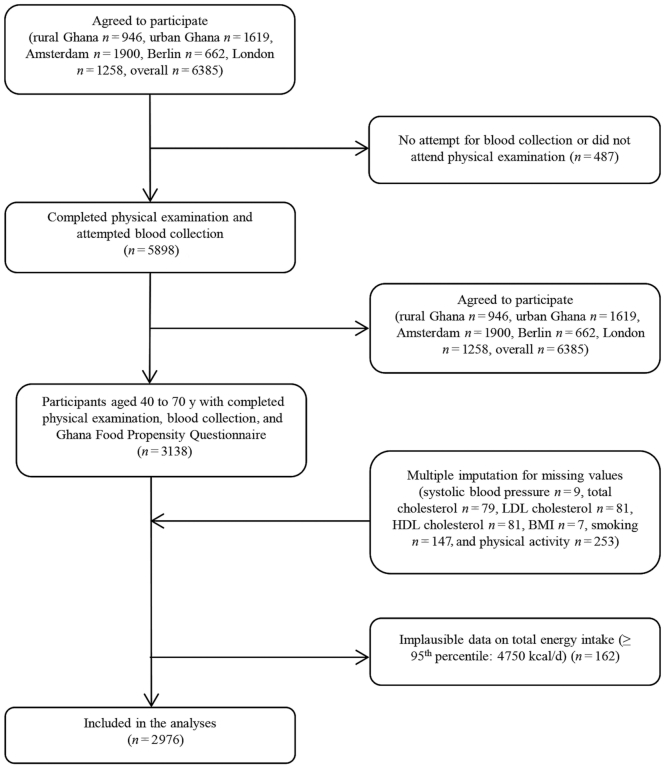
Selection of participants. Out of 5898 participants with completed physical examination and attempted blood collection 3131 were aged 40–70 y and had completed the Ghana food propensity questionnaire; 2976 participants were finally included, after multiple imputation of missing values and removal of implausible data on total energy intake.

All RODAM study participants aged 40 to 70 y were included in the current analysis. Subjects with a history of clinical atherosclerotic CVD (ASCVD) (*n* = 253) were excluded. Missing data (systolic blood pressure (BP) *n* = 9, serum total cholesterol *n* = 79, serum LDL cholesterol *n* = 81, serum HDL cholesterol *n* = 81, BMI (kg/m^2^) *n* = 7, smoking *n* = 147, and physical activity *n* = 253) were imputed. Five imputed datasets were created through regression-based multiple imputations employing the variables used in the main models. This involved multiple imputations by chained equations, using separate conditional univariate imputation models specified for each incomplete variable (28). This resulted in a final sample size of 2976 after the removal of 162 participants with implausible data on total energy intake (≥95^th^ percentile: 4,750 kcal/d) (**Figure 1**).

### Measurements

Trained study personnel performed all measurements with validated devices according to standardized operating procedures across all study sites. Fasting venous blood samples were collected, manually processed, and immediately aliquoted, and then temporarily stored at the local research location at −20°C. The samples were then transported to the respective local laboratories for registration and storage at −80°C and were subsequently transported to Berlin, Germany, for biochemical analysis to avoid intra-laboratory variability. Serum total cholesterol, serum HDL cholesterol, and serum LDL cholesterol were determined using the ABX Pentra 400 chemistry analyzer (HORIBA ABX). T2D was defined as fasting glucose ≥7.0 mmol/L or reported current use of medication prescribed to treat diabetes, or self-reported diabetes (29).

BP was measured three times using a validated semi-automated device (The Microlife WatchBP home) with appropriate cuffs in a sitting position after at least 5 min rest. The mean of the last two measurements was used in the analysis. Weight was measured twice in light clothing and without shoes with SECA 877 scales to the nearest 0.1 kg. Height was also measured twice without shoes with a portable stadiometer (SEC 217) to the nearest 0.1 cm. BMI was calculated as weight in kilograms (kg) divided by height in meters squared (m^2^). Overweight and obesity were defined as BMI ≥25 to <30 and ≤30, respectively (30).

### Questionnaire-based interviews

Interviews were conducted by a trained research assistant or self-administration of a paper questionnaire or digital online version depending on the preference of the participant (27). Physical activity was assessed using the WHO STEPwise approach to chronic disease risk factor Surveillance (STEPS) Questionnaire (31). Socio-demographic information included age, sex, and educational level, which was recorded as: never been to school or elementary school, lower vocational schooling or lower secondary schooling, intermediate vocational schooling or intermediate/higher secondary schooling, and higher vocational schooling or university. Use of antihypertensive medication was assessed based on a ‘Yes’ or ‘No’ response to the question ‘Do you use any antihypertensive medication, including combinations?’ Smoking status was based on either a ‘Yes’, ‘No, but I used to smoke’, or ‘No, I've never smoked’ response to the question ‘Do you smoke at all?’.

### Dietary assessment and dietary patterns

We used previously identified DPs derived in the RODAM study as exposure variables. Details of the pattern analysis have been published elsewhere (21). Briefly, the Ghana-food propensity questionnaire (Ghana-FPQ) queried the usual intake frequencies of 134 food items in predefined portion sizes over the past 12 mo. Energy intake was calculated using the latest versions of the West African Food Composition Table and the German Nutrient Database (BLS 3.01, 2010) (21). For the DP analysis, the food items were further collapsed into 30 food groups according to their similarities in nutrient composition and culinary use. Exploratory DPs were derived by principal component analysis (PCA) with an orthogonal rotation (VARIMAX) to identify underlying pattern scores that explained the maximum invariance of these 30 food groups. Every participant was assigned a pattern score for each DP to be ranked according to the degree of pattern adherence (21). Three DPs were identified, explaining 29% of the total variance in food intake. The identified DPs and their rotated factor loadings are presented in **Supplemental Table 1** (21).

The first factor, ‘mixed' pattern, explained 14.4% of the total variance in food intake and was characterized by high intakes of whole grain cereals, sweet spreads, dairy products, potatoes, poultry, vegetables, coffee and tea, sodas and juices, margarine, olive oil, and condiments, and by a low intake of palm oil and vegetarian mixed dishes. The second factor, ‘rice, pasta, meat, and fish’ pattern, was characterized by high intakes of dairy products, processed meat, red meat, legumes, eggs, fish, rice and pasta, meaty mixed dishes, and cakes, sweets and condiments. It accounted for 8.8% of the total variance in food intake. The third factor, called ‘roots, tubers, and plantain’ pattern, accounted for 5.7% of the total variance in food intake and was characterized by high intakes of refined cereals, fermented maize products, legumes, palm oil, and nuts (21). Participants in this study were ranked according to quintiles of adherence to the food patterns.

### Estimated 10-y CVD risk

The outcome variable was 10-y ASCVD risk, predicted by the Pooled Cohort Equations (PCE) for African-American men and women (26). This model combines age, sex, total cholesterol, HDL cholesterol, systolic BP, use of antihypertensive medication, diagnosed with T2D, and smoking to obtain the predicted 10-y ASCVD risk in people without pre-existing CVD (26). In their updated clinical practice guidelines for the treatment of blood cholesterol to reduce ASCVD, the American College of Cardiology (ACC) and AHA recommended the PCE as a novel tool to estimate 10-y ASCVD risk (32). The guidelines provide a strong recommendation (Class I, Level of Evidence: A) for consideration of statin treatment in individuals with a predicted 10-y ASCVD risk ≥7.5% and a moderate recommendation (Class IIa, Level of Evidence: B) in individuals with a predicted 10-y ASCVD risk of 5% to <7.5%. A predicted 10-y ASCVD risk ≥7.5% was considered as ‘elevated’ risk based on prior work by Goff et al. (26).

### Statistical analysis

General characteristics are summarized as percentages for categorical variables, mean ± SD for normally distributed continuous variables and as median (IQR) for nonnormally distributed variables. The distributions of socio-demographic characteristics, CVD risk factors, and mean intake of food groups (g/d) across quintiles of DPs and respective *P* values for trend were assessed using Pearson chi-square for proportions and one-factor ANOVA for continuous variables. For continuous variables, the *P* value for trend was calculated by Pearson correlations. Prevalence ratios (PR) and 95% CI were calculated using Poisson regression with robust variance (33) to assess the associations between the dietary patterns (per quintile) and predicted high 10-y risk of ASCVD. In addition, the associations with predicted high 10-y ASCVD risk were calculated per 1 SD increase of the DPs. β-coefficients, 95% CIs, and *P* values of estimated CVD risk per 1 SD increase of serum HDL cholesterol, serum LDL cholesterol, serum total cholesterol, and systolic BP were calculated by linear regression, whereas the PR and 95% CI for 1 SD increase in the prevalence of T2D were calculated by Poisson regression with robust variance. Three models were constructed: Model 1 adjusted for age, sex, education, and length of stay in Europe (only for the migrant sample); Model 2: model 1 together with BMI; and Model 3: model 2 together with total energy intake expressed in kcal/d and physical activity in metabolic equivalent of tasks (METs-h/wk). Apart from age (adjusted as a categorical variable) and sex, other variables included in the CVD risk score were not adjusted to avoid over-adjusting. The analyses were stratified by study site to investigate the contextual differences in DPs and 10-y CVD risk. For all statistical tests, a two-sided *P* value of <0.05 was considered statistically significant. Data were analyzed using SPSS version 25 (34).

## Results

The socio-demographic characteristics and lipid profile of participants by study site are presented in **Supplemental Table 2**. Briefly, the mean age (±SD) of the study participants was 52 y (±7.6) and the participants from rural Ghana were slightly older than those from urban Ghana and Europe. Educational level was highest in Europe and lowest in rural Ghana. Mean systolic BP and serum HDL cholesterol were significantly higher in Europe than in urban and rural Ghana. Mean serum LDL and total cholesterol were significantly higher in urban Ghana than all other sites. The prevalence of T2D was higher in Europe (14.1%) than in rural Ghana (6.1%). The prevalences of overweight and obesity were also highest in Europe and lowest in rural Ghana.

The distribution of socio-demographic characteristics by quintiles of the DPs is shown in **Tables 1**–**3**. In Europe, the majority of participants who adhered to the ‘mixed’ and ‘roots, tubers, and plantain’ DPs were older whereas those who adhered to the ‘rice, pasta, meat, and fish’ DP were younger compared with those in the lowest quintile, Table 1. Adherence to the ‘mixed' DP was associated with higher reported total energy intake and longer duration of stay in Europe. Participants in the highest quintile of the ‘rice, pasta, meat, and fish’ DP had a lower proportion of antihypertensive usage and T2D, higher concentrations of serum HDL cholesterol, higher energy intake, longer duration of stay in Europe, and lower predicted 10-y ASCVD risk compared with counterparts in the lowest quintile.

**TABLE 1:** Distribution of socio-demographic and cardiovascular disease risk factors by quintiles of dietary pattern scores in Europe^1^

	Mixed dietary pattern				Rice, pasta, meat, and fish dietary pattern				Root, tubers, and plantain pattern			
Variables	Q1 *n* = 278	Q3 *n* = 279	Q5 *n* = 278	*P*-trend	Q1 *n* = 278	Q3 *n* = 279	Q5 *n* = 278	*P*-trend	Q1 *n* = 278	Q3 *n* = 279	Q5 *n* = 278	*P*-trend
Age, y	50 ± 6.7	51 ± 7.0	52 ± 7.2	0.001	52 ± 7.3	51 ± 7.0	50 ± 6.9	<0.001	50 ± 6.2	50 ± 6.9	52 ± 7.1	<0.001
Sex (male), %	47.1	38.4	46.0	0.168	45.7	39.8	50.4	0.076	47.5	43.4	37.4	0.086
Education^2^, %	—	—	—	<0.001	—	—	—	0.027	—	—	—	0.010
Never or elementary	23.8	26.5	15.9		25.6	27.7	16.7		26.8	31.1	17.5	
Low	43.5	39.7	37.0		35.9	38.7	40.6		37.2	37.0	38.7	
Intermediate	22.7	22.8	32.6		26.7	24.4	30.1		23.8	21.6	27.5	
Higher vocational or tertiary	10.0	11.0	14.5		11.9	9.2	12.7		12.3	10.3	16.4	
Systolic blood pressure, mmHg	137 ± 18.1	138 ± 16.9	137 ± 17.1	0.973	139 ± 15.8	138 ± 18.4	138 ± 18.5	0.501	137 ± 16.1	136 ± 17.6	138 ± 16.8	0.098
Use of antihypertensives, %	33.5	39.4	38.1	0.272	41.7	40.5	33.1	0.022	35.6	33.7	44.2	0.076
Serum total cholesterol, mmol/L	5.3 ± 1.2	5.1 ± 1.0	5.2 ± 1.1	0261	5.1 ± 1.1	5.1 ± 1.3	5.2 ± 1.1	0.056	5.2 ± 1.2	5.1 ± 1.2	5.2 ± 1.1	0.475
Serum HDL cholesterol, mmol/L	1.4 ± 0.4	1.4 ± 0.3	1.5 ± 0.4	0.053	1.4 ± 0.3	1.4 ± 0.3	1.5 ± 0.3	0.027	1.4 ± 0.3	1.4 ± 0.3	1.4 ± 0.3	0.947
Serum LDL cholesterol, mmol/L	3.4 ± 1.0	3.3 ± 0.9	3.3 ± 0.9	0.154	3.3 ± 1.0	3.3 ± 1.0	3.3 ± 0.9	0.283	3.4 ± 1.1	3.3 ± 1.0	3.3 ± 1.0	0.632
Smoking, %	3.3	5.1	6.5	0.341	5.0	2.2	6.5	0.057	5.0	2.9	5.0	0.430
Type 2 diabetes mellitus, %	13.3	16.8	13.7	0.647	19.4	14.3	12.2	0.033	13.7	15.8	15.8	0.403
BMI, kg/m^2^	28.8 ± 4.2	29.8 ± 4.7	28.6 ± 4.5	0.323	29.2 ± 4.7	29.6 ± 5.0	28.4 ± 5.0	0.099	29.5 ± 4.8	29.0 ± 4.6	28.9 ± 4.8	0.276
Physical activity, METs-h/wk^3^, median (25^th^, 75^th^ percentile)	52.0 (8.3, 168)	62.0 (17.3, 184)	95.0 (26.3, 198)	0.154	60.0 (10.0, 175)	84.0 (16.0, 203)	92.5 (17.3, 196)	0.073	72.0 (11.9, 196)	82.0 (20.0, 197)	61.0 (14.0, 156.0)	0.131
Total energy intake, kcal/d	2,306 ± 832	2,513 ± 858	3,157 ± 806	<0.001	2,270 ± 896	2,509 ± 833	3,270 ± 789	<0.001	2,553 ± 858	2,346 ± 841	3,065 ± 853	<0.001
Length of stay in Europe, y	17.1 ± 8.7	19.7 ± 8.7	21.2 ± 9.6	<0.001	20.6 ± 9.0	19.5 ± 9.4	18.5 ± 9.6	0.001	18.4 ± 8.6	19.2 ± 8.4	21.4 ± 10.2	<0.001
High ASCVD risk (≥ 7.5%), %	34.2	33.3	38.6	0.505	43.1	39.8	34.2	0.018	31.3	33.7	42.1	0.064

^1^Data are percentages, means ± SDs, or median (25^th^, 75^th^ percentiles). *P*-trend was calculated for quantitative variables (as continuous variables) and by the chi-square test for categorical variables. ASCVD, atherosclerotic cardiovascular disease; MET, metabolic equivalent of task.

^2^Level of education: Elementary = primary or basic school education; Low = lower/junior secondary; Intermediate = intermediate vocational schooling or intermediate/higher secondary schooling, sixth form, or college; Higher vocational or tertiary = tertiary including university or polytechnic.

^3^Physical activity was assessed using the WHO STEPwise approach to the chronic disease risk factor Surveillance (STEPS) Questionnaire (31).

In urban Ghana, adherence to the ‘mixed’ and ‘rice, pasta, meat, and fish’ DPs was associated with a higher BMI, whereas the ‘roots, tubers, and plantain’ DP was associated with a lower BMI, Table 2. Participants who adhered to the ‘mixed’ DP were more educated and had reduced physical activity. Adherence to the ‘rice, pasta, meat, and fish’ DP was associated with being younger, reduced systolic BP, lower antihypertensive usage, and overall lower predicted 10-y ASCVD risk. Participants in rural Ghana who adhered to the ‘rice, pasta, meat, and fish’ DP were younger, and had a higher BMI and lower 10-y ASCVD risk, Table 3.

**TABLE 2:** Distribution of socio-demographic and cardiovascular disease risk factors by quintiles of dietary pattern scores in urban Ghana^1^

	Mixed dietary pattern				Rice, pasta, meat, and fish dietary pattern				Root, tubers, and plantain pattern			
Variables	Q1 *n* = 188	Q3 *n* = 189	Q5 *n* = 189	*P*-trend	Q1 *n* = 188	Q3 *n* = 189	Q5 *n* = 189	*P*-trend	Q1 *n* = 188	Q3 *n* = 189	Q5 *n* = 189	*P*-trend
Age, y	52 ± 7.9	52 ± 8.1	51 ± 7.5	0.452	55 ± 8.1	52 ± 7.3	49 ± 7.4	<0.001	53 ± 7.9	52 ± 7.4	51 ± 7.6	0.022
Sex (male), %	28.7	31.7	27.0	0.842	21.8	29.1	38.1	0.002	27.7	27.0	33.9	0.307
Education^2^, %	—	—	—	0.001	—	—	—	<0.001	—	—	—	0.003
Never or elementary	42.7	53.5	42.5		63.9	47.6	34.9		54.3	51.4	46.6	
Low	47.6	29.7	37.9		21.8	37.0	48.1		28.3	40.4	34.9	
Intermediate	5.9	14.6	10.9		9.6	11.6	12.7		12.2	4.9	12.2	
Higher vocational or tertiary	3.8	2.2	8.6		4.8	3.7	4.2		3.7	3.3	6.3	
Systolic blood pressure, mmHg	133 ± 21.0	133 ± 21.2	130 ± 17.4	0.116	135 ± 22.4	133 ± 20.8	127 ± 17.9	0.004	132 ± 21.2	132 ± 20.1	131 ± 21.1	0.497
Use of antihypertensives, %	14.4	16.4	16.9	0.704	19.7	17.5	7.9	0.021	11.7	18.5	14.3	0.332
Serum total cholesterol, mmol/L	5.5 ± 1.3	5.5 ± 1.2	5.5 ± 1.0	0.370	5.3 ± 1.1	5.6 ± 1.2	5.4 ± 1.2	0.617	5.5 ± 1.1	5.3 ± 1.1	5.4 ± 1.1	0.310
Serum HDL cholesterol, mmol/L	1.3 ± 0.4	1.3 ± 0.3	1.3 ± 0.3	0.845	1.3 ± 0.4	1.3 ± 0.3	1.3 ± 0.3	0.413	1.3 ± 0.3	1.3 ± 0.3	1.3 ± 0.3	0.801
Serum LDL cholesterol, mmol/L	3.7 ± 1.0	3.7 ± 0.9	3.7 ± 0.8	0.557	3.5 ± 1.0	3.8 ± 1.1	3.6 ± 0.9	0.506	3.7 ± 1.0	3.5 ± 1.0	3.6 ± 1.0	0.631
Smoking, %	0.5	0.0	0.5	0.337	0.0	1.1	1.1	0.514	0.0	0.5	2.1	0.216
Type 2 diabetes mellitus, %	15.4	13.2	9.5	0.520	14.9	11.1	8.5	0.081	14.4	16.4	9.0	0.199
BMI, kg/m^2^	26.7 ± 4.9	27.4 ± 5.3	28.7 ± 5.8	0.003	26.5 ± 5.2	27.3 ± 4.8	28.1 ± 5.1	<0.001	27.6 ± 5.4	27.1 ± 5.3	26.8 ± 4.9	0.028
Physical activity, METs-h/wk^3^, median (25^th^, 75^th^ percentile)	120 (24.0, 205)	30.0 (0.0, 115)	42.0 (9.0, 112)	<0.001	28.0 (0.0, 133)	70.0 (4.5, 167)	84.0 (25.5, 172)	0.001	80.0 (0, 174)	48.0 (4.0, 148)	60.0 (14.0, 126.0)	0.310
Total energy intake, kcal/d	2,087 ± 561	2,113 ± 643	2,701 ± 688	<0.001	1,792 ± 589	2,138 ± 496	2,857 ± 617	<0.001	1,890 ± 581	2,130 ± 544	2,769 ± 669	<0.001
High ASCVD risk (≥ 7.5%), %	36.7	34.4	25.9	0.142	42.6	31.7	20.1	<0.001	37.2	31.2	29.6	0.280

^1^Data are percentages, means ± SDs, or median (25^th^, 75^th^ percentiles). *P*-trend was calculated for quantitative variables (as continuous variables) and by the chi-square test for categorical variables. ASCVD, atherosclerotic cardiovascular disease; MET, metabolic equivalent of task.

^2^Level of education: Elementary = primary or basic school education; Low = lower/junior secondary; Intermediate = intermediate vocational schooling or intermediate/higher secondary schooling, sixth form, or college; Higher vocational or tertiary = tertiary including university or polytechnic.

^3^Physical activity was assessed using the WHO STEPwise approach to the chronic disease risk factor Surveillance (STEPS) Questionnaire (31).

**TABLE 3:** Distribution of socio-demographic and cardiovascular disease risk factors by quintiles of dietary pattern scores in rural Ghana^1^

	Mixed dietary pattern				Rice, pasta, meat, and fish dietary pattern				Root, tubers, and plantain pattern			
Variables	Q1 *n* = 127	Q3 *n* = 128	Q5 *n* = 128	*P*-trend	Q1 *n* = 127	Q3 *n* = 128	Q5 *n* = 128	*P*-trend	Q1 *n* = 127	Q3 *n* = 128	Q5 *n* = 128	*P*-trend
Age, y	54 ± 9.4	54 ± 8.4	53 ± 8.9	0.937	56 ± 9.1	54 ± 8.9	51 ± 8.2	<0.001	53 ± 8.9	54 ± 8.1	52 ± 9.1	0.011
Sex (male), %	30.7	36.7	36.7	0.539	33.9	33.6	38.3	0.774	33.1	32.8	36.7	0.427
Education^2^, %	—	—	—	0.282	—	—	—	0.073	—	—	—	0.558
Never or elementary	64.4	57.8	62.5		66.1	65.6	59.4		66.1	57.8	64.1	
Low	31.4	31.3	25.0		22.8	30.5	26.6		21.3	31.3	27.3	
Intermediate	3.4	6.3	9.4		10.2	2.3	10.2		8.7	8.6	4.7	
Higher vocational or tertiary	0.8	4.7	3.1		0.8	1.6	3.9		3.9	2.3	1.6	
Systolic blood pressure, mmHg	127 ± 20.9	130 ± 21.5	129 ± 21.9	0.459	132 ± 24.8	128 ± 19.6	128 ± 21.3	0.228	128 ± 21.2	130 ± 20.1	128 ± 21.1	0.981
Use of antihypertensives, %	15.7	14.1	3.9	0.007	15.0	10.2	6.3	0.092	14.2	3.9	7.0	0.008
Serum total cholesterol, mmol/L	4.7 ± 1.2	4.6 ± 1.1	4.2 ± 1.1	0.639	4.6 ± 1.1	4.7 ± 1.2	4.9 ± 1.2	0.083	4.6 ± 1.1	4.6 ± 1.1	4.6 ± 1.1	0.594
Serum HDL cholesterol, mmol/L	1.2 ± 0.4	1.1 ± 0.4	1.2 ± 0.4	0.306	1.2 ± 0.4	1.2 ± 0.4	1.2 ± 0.3	0.053	1.2 ± 0.4	1.1 ± 0.4	1.2 ± 0.3	0.058
Serum LDL cholesterol, mmol/L	3.0 ± 0.9	2.9 ± 0.9	2.9 ± 0.9	0.760	2.9 ± 1.0	3.0 ± 1.0	3.1 ± 0.9	0.225	2.9 ± 1.0	2.9 ± 1.0	2.9 ± 1.0	0.244
Smoking, %	1.6	2.3	2.3	0.700	3.1	0.8	1.6	0.310	2.4	0.0	3.1	0.121
Type 2 diabetes mellitus, %	7.1	6.3	4.7	0.936	11.0	6.3	4.7	0.063	7.9	7.8	5.5	0.754
BMI, kg/m^2^	22.9 ± 4.6	22.5 ± 4.4	22.7 ± 4.6	0.479	21.8 ± 4.3	22.4 ± 4.0	23.9 ± 5.1	0.001	23.0 ± 4.1	22.7 ± 4.3	21.8 ± 4.3	0.078
Physical activity, METs-h/wk^3^, median (25^th^, 75^th^ percentile)	106 (57.6, 200)	89.0 (27.0, 171)	80.0 (25.5, 176)	0.127	90.0 (30.0, 182)	90.0 (36.0, 168)	40.5 (99.5, 189)	0.059	104 (54.0, 2001)	82.5 (24,7, 160)	100 (38, 218)	0.258
Total energy intake, kcal/d	2,252 ± 636	2,346 ± 678	3,141 ± 848	<0.001	2,155 ± 818	2,388 ± 712	3,166 ± 746	<0.001	1,897 ± 537	2,366 ± 542	6,550 ± 669	<0.001
High ASCVD risk (≥ 7.5%), %	32.3	32.8	26.6	0.112	44.1	33.6	25.8	0.004	31.5	31.3	28.1	0.695

^1^Data are percentages, means ± SDs, or median (25^th^, 75^th^ percentiles). *P*-trend was calculated for quantitative variables (as continuous variables) and by the chi-square test for categorical variables. ASCVD, atherosclerotic cardiovascular disease; MET, metabolic equivalent of task.

^2^Level of education: Elementary = primary or basic school education; Low = lower/junior secondary; Intermediate = intermediate vocational schooling or intermediate/higher secondary schooling, sixth form, or college; Higher vocational or tertiary = tertiary including university or polytechnic.

^3^Physical activity was assessed using the WHO STEPwise approach to the chronic disease risk factor Surveillance (STEPS) Questionnaire (31).

**FIGURE 2:**
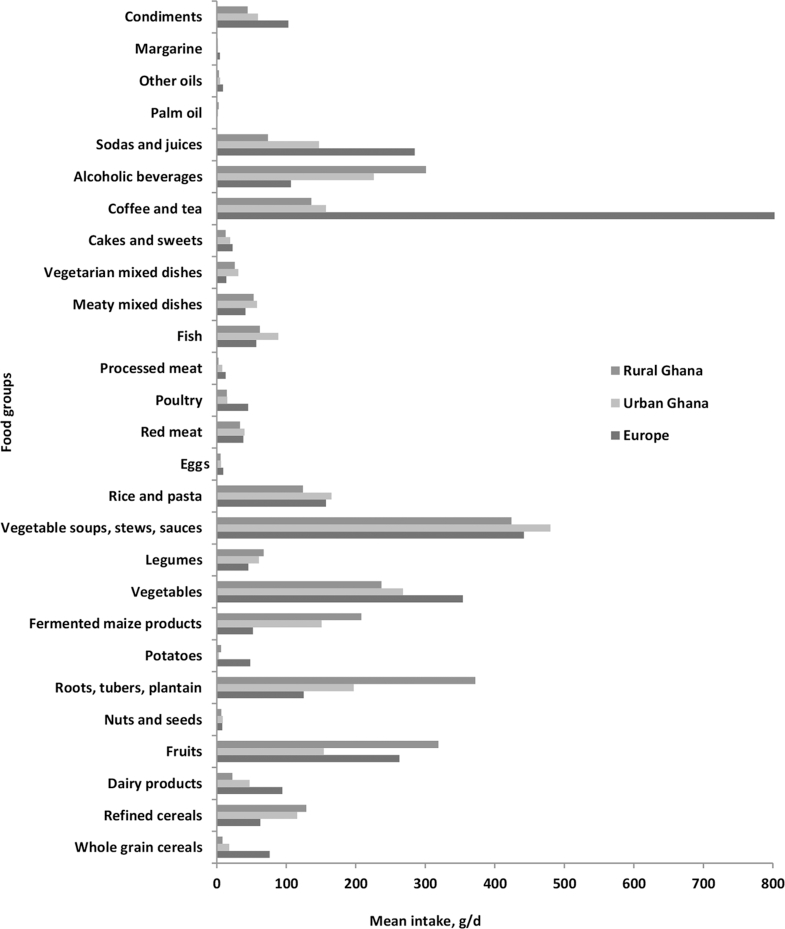
The mean intake of food groups by Ghanaians in the RODAM study. The mean intake of foods differed significantly between Europe, urban Ghana, and rural Ghana.

The mean intake of food groups or components of the DPs differed significantly by study sites, as detailed in **Figure 2**. For instance, the mean intake of whole grains, bread and cereals, refined cereals, sweet spreads, dairy products, vegetables, eggs, poultry, coffee and tea, olive oil, and condiments was highest in Europe and lowest in rural Ghana. The mean intake of vegetables, stews, sauces, red meat, fish, and meaty mixed dishes was highest among Ghanaians in urban Ghana, whereas the mean intake of roots, tubers, plantain, legumes, and palm oil was highest in rural Ghana.

The distribution of food groups across the quintiles of the DPs is also shown in **Supplemental Tables 3–5**. Generally, the distribution of food intake across quintiles of DPs differed by study site. For instance, among Ghanaians in Europe, the mean intake of whole grains increased across quintiles of ‘mixed’ and ‘root, tubers, and plantain’ DPs and decreased across quintiles of ‘rice, pasta, meat, and fish’ DPs. The mean intake of condiments also increased across ‘mixed’ and ‘rice, pasta, meat, and fish’ DPs and decreased across quintiles of ‘rice, pasta, meat, and fish’ DPs. The mean intake of food groups varied across quintiles of DPs in all study sites.

The associations between DPs and modifiable CVD risk factors in the PCE model are presented in **Supplementary Table 6**. In Europe, the ‘mixed’ DP was directly associated with serum HDL cholesterol after adjustment for possible confounders. An inverse association with T2D was observed but the effect was attenuated after adjustment for possible confounders. In urban Ghana, adherence to the ‘mixed’ DP was inversely associated with systolic BP and T2D in the crude models whereas the ‘rice, pasta, meat, and fish’ DP was inversely associated with T2D and reduced systolic BP in the crude models but not in the fully adjusted models. The ‘rice, pasta, meat, and fish’ DP was associated with increased serum total cholesterol in rural Ghana.

The associations between DPs and predicted 10-y risk of ASCVD are presented in **Tables 4**–**6**. The ‘mixed’ and ‘root, tubers, and plantain’ DPs were associated with elevated estimated 10-y ASCVD risk in the migrant population with attenuation of the effect in the adjusted models, see Table 4. In urban Ghana, the PR for elevated 10-y ASCVD risk was 0.71 times lower in the highest quintile compared with the lowest quintile of the ‘mixed’ DP (PR: 0.71; 95% CI: 0.52, 0.96, *P*-trend = 0.57). Per 1 SD increase of this DP, the estimated 10-y elevated ASCVD risk decreased by 11% (PR: 0.89; 95% CI: 0.80, 0.98) in model 1. The association remained consistent after further adjustment in the full model, see Table 5. Similarly, adherence to the ‘rice, meat, pasta, and fish’ DP was associated with lower prevalence of 10-y CVD risk, but the effect was attenuated in the final model. In rural Ghana, the PR of predicted 10-y ASCVD risk increased with adherence to the ‘root, tubers, and plantain’ DP. The ‘mixed’ and ‘rice, pasta, meat, and fish’ DPs were not associated with 10-y CVD risk in rural Ghana, see Table 6.

**TABLE 4:** Prevalence ratios (95% CIs) of predicted 10-y ASCVD risk by dietary pattern in Europe, the RODAM study^1^

	PR (95% CI)										PR per 1 SD increase (95% CI)
	Q1 (Ref.)	Q2		Q3		Q4		Q5			
Model		PR (95% CI)	*P* value	PR (95% CI)	*P* value	PR (95% CI)	*P* value	PR (95% CI)	*P* value	*P*-trend	
*Mixed dietary patterns*											
‘Elevated’ ASCVD risk/total	96/278	102/279		97/279		108/279		108/278			—
Crude	1.00	1.07 (0.85, 1.34)	0.556	1.01 (0.80, 1.27)	0.953	1.13 (0.91, 1.41)	0.267	1.14 (0.91, 1.42)	0.253	0.257	1.03 (0.97, 1.11)
Model 1	1.00	1.12 (0.92, 1.36)	0.265	1.02 (0.84, 1.23)	0.860	1.01 (0.84, 1.21)	0.930	0.98 (0.81, 1.17)	0.786	0.400	0.98 (0.93, 1.04)
Model 2	1.00	1.13 (0.93, 1.34)	0.225	1.01 (0.84, 1.23)	0.893	1.02 (0.85, 1.22)	0.846	1.00 (0.83, 1.20)	0.846	0.560	0.99 (0.94, 1.05)
Model 3	1.00	1.14 (0.93, 1.38)	0.206	1.04 (0.85, 1.26)	0.727	1.05 (0.87, 1.26)	0.634	1.05 (0.85, 1.28)	0.670	0.975	1.01 (0.95, 1.07)
*Rice, pasta, meat, and fish*											
‘Elevated’ ASCVD risk/total	120/278	101/279		113/279		82/279		97/278			—
Crude	1.00	0.82 (0.67, 1.01)	0.065	0.93 (0.76, 1.13)	0.470	0.67 (0.53, 0.85)	0.001	0.81 (0.66, 0.99)	0.047	0.011	0.91 (0.85, 0.98)
Model 1	1.00	0.89 (0.75, 1.07)	0.220	1.04 (0.89, 1.25)	0.579	0.86 (0.71, 1.04)	0.122	0.92 (0.77, 1.09)	0.315	0.157	0.97 (0.92, 1.03)
Model 2	1.00	0.90 (0.73, 1.06)	0.145	1.01 (0.85, 1.20)	0.888	0.85 (0.70, 1.02)	0.081	0.91 (0.77, 1.09)	0.301	0.153	0.97 (0.91, 1.02)
Model 3	1.00	0.91 (0.76, 1.09)	0.308	1.04 (0.87, 1.24)	0.689	0.87 (0.72, 1.05)	0.139	0.95 (0.78, 1.14)	0.579	0.287	0.98 (0.92, 1.04)
*Roots, tubers, and plantain*											
‘Elevated’ ASCVD risk/total	87/278	108/279		95/279		104/279		117/278		—	—
Crude	1.00	1.23 (0.98, 1.54)	0.082	1.08 (0.85, 1.37)	0.546	1.19 (0.95, 1.50)	0.138	1.34 (1.08, 1.68)	0.009	0.025	1.07 (1.01, 1.14)
Model 1	1.00	1.12 (0.93, 1.36)	0.247	0.97 (0.80, 1.18)	0.761	0.96 (0.80, 1.15)	0.642	1.14 (0.95, 1.37)	0.153	0.582	1.02 (0.96, 1.08)
Model 2	1.00	1.12 (0.94, 1.38)	0.196	1.00 (0.82, 1.22)	0.989	1.00 (0.83, 1.20)	0.957	1.20 (1.00, 1.44)	0.055	0.260	1.03 (0.98, 1.09)
Model 3	1.00	1.14 (0.95, 1.38)	0.201	1.00 (0.81, 1.22)	0.939	1.02 (0.84, 1.23)	0.876	1.23 (1.02, 1.48)	0.030	0.183	1.04 (0.99, 1.10)

^1^Model 1: Age (40–50 y, 50–60 y, 60–70 y), education (categorical), length of stay, y; Model 2: Model 1 + BMI; Model 3: Model 2 + total energy intake, kcal/d (quintiles), physical activity, METs-h/wk (quintiles). ASCVD, atherosclerotic cardiovascular disease; PR, prevalence ratio; Ref., referent.

**TABLE 5:** Prevalence ratios (95% CIs) of estimated 10-y ASCVD risk by dietary pattern in urban Ghana, the RODAM study^1^

	PR (95% CI)										PR per 1 SD increase (95% CI)
	Q1 (Ref.)	Q2		Q3		Q4		Q5			
Model		PR (95% CI)	*P* value	PR (95% CI)	*P* value	PR (95% CI)	*P* value	PR (95% CI)	*P* value	*P*-trend	
*Mixed dietary patterns*											
‘Elevated’ ASCVD risk/total	69/188	54/189		65/189		56/189		49/189			—
Crude	1.00	0.78 (0.58, 1.04)	0.094	0.94 (0.71, 1.23)	0.639	0.81 (0.61, 1.08)	0.147	0.71 (0.52, 0.96)	0.026	0.057	0.89 (0.80, 0.98)
Model 1	1.00	0.78 (0.61, 0.99)	0.044	0.88 (0.72, 1.09)	0.238	0.84 (0.66, 1.07)	0.161	0.74 (0.57, 0.96)	0.026	0.071	0.90 (0.82, 0.99)
Model 2	1.00	0.78 (0.62, 0.99)	0.048	0.89 (0.72, 1.10)	0.281	0.86 (0.67, 1.10)	0.229	0.72 (0.55, 0.94)	0.015	0.051	0.89 (0.82, 0.98)
Model 3	1.00	0.75 (0.59. 0.94)	0.014	0.83 (0.67, 1.02)	0.082	0.80 (0.63, 1.03)	0.082	0.70 (0.53, 0.93)	0.013	0.042	0.89 (0.81, 0.98)
*Rice, pasta, meat, and fish*											
‘Elevated’ ASCVD risk/total	80/188	69/189		60/189		46/189		38/189			—
Crude	1.00	0.86 (0.67, 1.10)	0.231	0.75 (0.57, 0.97)	0.031	0.57 (0.42, 0.77)	<0.001	0.47 (0.34, 0.66)	<0.001	<0.001	0.79 (0.71, 0.89)
Model 1	1.00	0.97 (0.80, 1.18)	0.749	0.89 (0.71, 1.12)	0.322	0.78 (0.60, 1.00)	0.051	0.70 (0.53, 0.93)	0.013	0.003	0.93 (0.84, 1.01)
Model 2	1.00	0.95 (0.78, 1.16)	0.606	0.86 (0.68, 1.08)	0.184	0.76 (0.59, 0.97)	0.030	0.67 (0.51, 0.88)	0.004	0.001	0.90 (0.82, 0.99)
Model 3	1.00	0.96 (0.79, 1.18)	0.711	0.89 (0.71, 1.12)	0.319	0.79 (0.61, 1.02)	0.075	0.72 (0.53, 0.99)	0.045	0.015	0.95 (0.85, 1.06)
*Roots, tubers, and plantain*											
‘Elevated’ ASCVD risk/total	70/188	51/189		59/189		57/189		56/189			—
Crude	1.00	0.73 (0.54, 0.98)	0.035	0.84 (0.63, 1.11)	0.220	0.81 (0.61, 1.08)	0.148	0.80 (0.60, 1.06)	0.120	0.270	0.97 (0.88, 1.07)
Model 1	1.00	0.84 (0.66, 1.05)	0.127	0.90 (0.72, 1.13)	0.351	0.87 (0.69, 1.09)	0.222	0.93 (0.73, 1.17)	0.516	0.588	1.01 (0.93, 1.09)
Model 2	1.00	0.84 (0.66, 1.06)	0.139	0.91 (0.73, 1.14)	0.432	0.88 (0.70, 1.11)	0.276	0.95 (0.75, 1.19)	0.632	0.723	1.02 (0.94, 1.10)
Model 3	1.00	0.85 (0.67, 1.08)	0.176	0.93 (0.74, 1.18)	0.561	0.92 (0.73, 1.17)	0.507	1.05 (0.80, 1.37)	0.726	0.672	1.08 (0.98, 1.18)

^1^Model 1: Age (40–50 y, 50–60 y, 60–70 y), sex, education (categorical); Model 2: Model 1 + BMI; Model 3: Model 2 + total energy intake, kcal/d (quintiles), physical activity, METs-h/wk (quintiles). ASCVD, atherosclerotic cardiovascular disease; PR, prevalence ratio; Ref., referent.

**TABLE 6:** Prevalence ratios (95% CIs) of estimated 10-y ASCVD risk by dietary pattern in rural Ghana, the RODAM study^1^

	PR (95% CI)										PR for per 1 SD increase (95% CI)
	Q1 (Ref.)	Q2		Q3		Q4		Q5			
Model		PR (95% CI)	*P* value	PR (95% CI)	*P* value	PR (95% CI)	*P* value	PR (95% CI)	*P* value	*P*-trend	
*Mixed dietary patterns*											
‘Elevated’ ASCVD risk/total	41/127	50/128		42/128		32/128		34/128			—
Crude	1.00	1.21 (0.89, 1.69)	0.261	1.02 (0.71, 1.45)	0.928	0.77 (0.52, 1.15)	0.201	0.82 (0.56, 1.21)	0.318	0.047	0.93 (0.82, 1.05)
Model 1	1.00	1.06 (0.82, 1.35)	0.673	0.92 (0.71, 1.19)	0.516	0.81 (0.60, 1.09)	0.156	0.83 (0.61, 1.13)	0.237	0.052	0.92 (0.83, 1.02)
Model 2	1.00	1.10 (0.86, 1.42)	0.451	0.94 (0.73, 1.21)	0.629	0.81 (0.60, 1.09)	0.159	0.84 (0.62, 1.13)	0.245	0.039	0.93 (0.82, 1.01)
Model 3	1.00	1.10 (0.86, 1.42)	0.449	0.94 (0.73, 1.21)	0.634	0.81 (0.60, 1.09)	0.157	0.84 (0.61, 1.16)	0.283	0.052	0.92 (0.83, 1.02)
*Rice, pasta, meat, and fish*											
‘Elevated’ ASCVD risk/total	56/127	36/128		43/128		31/128		33/128			—
Crude	1.00	0.64 (0.45, 0.90)	0.009	0.76 (0.56, 1.04)	0.088	0.55 (0.38, 0.79)	0.001	0.59 (0.41, 0.83)	0.003	0.002	0.81 (0.71, 0.93)
Model 1	1.00	0.81 (0.63, 1.05)	0.115	0.94 (0.73, 1.22)	0.539	0.90 (0.66, 1.23)	0.510	0.93 (0.70, 1.24)	0.628	0.767	0.98 (0.89, 1.08)
Model 2	1.00	0.81 (0.62, 1.04)	0.102	0.90 (0.69, 1.17)	0.419	0.87 (0.64, 1.19)	0.382	0.86 (0.65, 1.14)	0.293	0.385	0.96 (0.87, 1.06)
Model 3	1.00	0.81 (0.63, 1.05)	0.114	0.91 (0.70, 1.18)	0.471	0.89 (0.65, 1.21)	0.443	0.89 (0.65, 1.21)	0.465	0.590	0.97 (0.88, 1.08)
*Roots, tubers, and plantain*											
‘Elevated’ ASCVD risk/total	40/127	46/128		40/128		37/128		36/128			—
Crude	1.00	1.14 (0.81, 1.81)	0.454	0.99 (0.69, 1.43)	0.966	0.92 (0.63, 1.33)	0.653	0.89 (0.61, 1.30)	0.557	0.268	0.94 (0.83, 1.06)
Model 1	1.00	1.17 (0.89, 1.55)	0.256	1.08 (0.81, 1.45)	0.591	1.06 (0.82, 1.51)	0.713	1.11 (0.82, 1.51)	0.511	0.835	1.01 (0.91, 1.11)
Model 2	1.00	1.25 (0.95, 1.58)	0.118	1.18 (0.87, 1.58)	0.296	1.12 (0.81, 1.53)	0.516	1.23 (0.90, 1.67)	0.200	0.480	1.02 (0.93, 1.12)
Model 3	1.00	1.28 (0.97, 1.72)	0.079	1.26 (0.93, 1.71)	0.131	1.28 (0.91, 1.80)	0.164	1.60 (1.10, 2.32)	0.013	0.042	1.14 (1.01, 1.30)

^1^Model 1: Age (40–50 y, 50–60 y, 60–70 y), sex, education (categorical); Model 2: Model 1 + BMI; Model 3: Model 2 + total energy intake, kcal/d (quintiles), physical activity, METs-h/wk (quintiles). ASCVD, atherosclerotic cardiovascular disease; PR, prevalence ratio; Ref., referent.

## Discussion

### Key findings

This study assessed the association between dietary habits and predicted 10-y ASCVD risk among Ghanaian migrant and home populations. We found similar levels of CVD risk reduction for the ‘mixed’ and ‘rice, pasta, meat, and fish’ DPs. Adherence to the ‘mixed’ and ‘rice, pasta, meat, and fish’ patterns was associated with a lower predicted 10-y risk of ASCVD in urban Ghana. The ‘roots, tubers, and plantain’ DP was associated with a higher predicted 10-y ASCVD risk in rural Ghana. The ‘mixed’ DP is characterized by a high intake of whole grain cereals, dairy products, poultry, potatoes, vegetables, margarine, olive oil, and condiments, whereas the ‘rice, pasta, meat, and fish’ DP was characterized by a high intake of dairy products, processed meat, red meat, legumes, eggs, fish, rice, and pasta, meaty mixed dishes, and cakes, sweets, and condiments. The ‘roots, tubers, and plantain’ was characterized by a high intake of refined cereals, fruits, fermented maize products, roots, tubers, plantain, and palm oil.

### Interpretation of findings

The overall influence of diet on multifactorial health outcomes may not be adequately captured by the use of single nutrients, thereby necessitating the use of DPs to fully understand the influence of diet on CVD risk (14). Previous work identified three DPs among Ghanaian adults (21). Adherence to a ‘mixed’ DP increased with urbanization and increased level of education. The ‘roots, tubers, and plantain’ DP was consumed at all study sites and in Europe, consumption increased with length of stay in Europe.

Although the ‘rice, pasta, meat, and fish’ and ‘mixed’ DPs were associated with a higher BMI in both Europe and urban Ghana, they were generally favorable for improved cardiovascular health, with adherence associated with higher serum HDL cholesterol, lower prevalence of T2D, and lower systolic BP. Recent analyses within this population found an inverse relation between adherence to the ‘rice, pasta, meat, and fish’ DP and T2D (15, 22). Other studies have also identified significant associations between DPs rich in legumes, poultry, fish, and whole grains and reduced risk of hypertension in Cameroon (17), and with T2D and several biomarkers for reduced cardiovascular disease risk elsewhere (35, 36). There is however no doubt that individual food choices, portion sizes, and general perception of food play a major role in the resulting CVD outcomes among different individuals.

Adherence to the ‘mixed’ and ‘rice, pasta, meat, and fish’ DPs was associated with a lower estimated 10-y ASCVD risk among Ghanaian adult populations, especially in urban Ghana. An inverse association between adherence to a ‘mixed-modern’ DP and metabolic syndrome has been found in an urban Samoan population undergoing a nutrition transition (37). Similarly, the ‘mixed’ and ‘rice, pasta, meat, and fish’ DPs were characterized by a high intake of some foods that are generally beneficial for cardiovascular health (38–40). The consumption of legumes, for instance, has been shown to improve cholesterol and BP, and to be protective against T2D (38, 39, 41). Although evidence of the benefits of legumes in the management of CVD is inconclusive, an inverse association between the consumption of legumes and coronary artery disease has been observed (40). The benefits of fish consumption in CVD reduction has also been reported in large epidemiological studies from Europe and North America (42).

Adherence to the ‘roots, tubers, and plantain’ DP was associated with a higher prevalence of predicted 10-y ASCVD risk in rural Ghana. Adherence to a more ‘traditional’ Ghanaian carbohydrate-dense DP is associated with T2D (16). Food components of the ‘roots, tubers, and plantain’ DP constitute the traditional Ghanaian staple foods and fruits, which are carbohydrate dense with a moderate to high glycemic index (GI) (43). Boiling, which is the main preparatory method for staple foods, further increases the GI through increased gelatinization and glucose response (44). Limited relevant research shows that a high GI diet unfavorably affects CVD risk (45, 46).

Findings from this study show significant contextual differences in dietary habits and food intake, resulting in a differential association between DPs and 10-y ASCVD risk among the various sites studied. Food availability and food choices among Ghanaian populations in different settings could partly explain the contextual differences in DPs and CVD risk. Dietary habits depend on the social and cultural environment, accessibility of foods, migration context, and food beliefs/perceptions (20). Ghanaians in Europe experience a ‘double’ nutrition transition, with an acculturation to a more ‘Western’ DP during the early years of migration. However, there is a return to traditional Ghanaian diets as a result of the general availability of foods from African countries to the European market (47), while still adopting some key features of the European food culture (20). We observed a reduced adherence to the ‘rice, pasta, meat, and fish’ DP but increased adherence to the ‘mixed’ and ‘roots, tubers, and plantain’ DPs with increasing length of stay in Europe. The intake of some ‘unhealthy’ components of the ‘mixed’ DP such as sweet spreads, sodas, and juices was relatively higher, possibly accounting for the high predicted 10-y ASCVD associated with adherence to this DP.

Ghanaians in urban Ghana also experience a nutrition transition, with a shift to a more westernized diet pattern (18, 48). This could mean exposure to a dietary variety including access to different sources of animal and food protein, legumes, and vegetables, which are not available in the traditional ‘root, tubers, and plantain’ DP. Most families in urban Ghana acquire most of their foods through purchasing (49). There are, therefore, many variations in household and individual diets in urban Ghana resulting in differences in risk of CVD, as food choices and access would depend on the level of education, income, and perceptions regarding diet and health. The main source of household food in rural Ghana is from the household's own food production, with less variety in comparison with urban Ghana (50). Previously, the ‘roots, tubers, and plantain’ DP showed weak correlations with between- and within-food group varieties (22) and this might also partly account for the poor beneficial effect of this DP on CVD risk (16).

The study of the effect of dietary patterns on predicted ASCVD risk provides insight into the dietary habits of high-risk groups in the population. The contextual differences in food intake, DPs, and CVD risk show how food availability and socio-economic backgrounds influence food choices and dietary diversity with a resultant influence on cardiovascular health. However, further research into dietary perceptions, food choices, and portion sizes will be needed to understand how these dietary habits influence ASCVD risk for dietary recommendations for this population as a means of CVD prevention.

### Strengths and limitations

The RODAM study, conducted among a population of Ghanaians with the same ancestry, provides a unique opportunity to assess the dietary patterns of Ghanaian populations living in different settings in Europe and Africa. This study provides important evidence on the influence of dietary habits on ASCVD risk among sub-Saharan African populations living in industrialized cities in Europe and their home country counterparts. However, given its cross-sectional nature, the correlation of predicted ASCVD risk with incident CVD events requires confirmation from prospective studies as the PCE risk algorithms used in predicting CVD in this study have not yet been validated for sub-Saharan African populations. Individual and environmental factors such as diet quality (variety, diversity), food preparation, access, and proximity to food sources that were not explored in this study are important factors that influence dietary habits and health and warrant further investigations.

The culture-specific, semi-quantitative FFQ demonstrated feasibility and good acceptance within the RODAM study (21). Generally, the use of an FFQ could be associated with measurement error and recall bias with, e.g., respondents giving socially desirable answers in relation to foods perceived as ‘healthy’ or ‘unhealthy’. Major DPs in this study were identified through PCA. Choices relating to consolidation of food items into food groups, the number of factors to extract, and the labeling of the components (14, 51, 52) could affect the reproducibility of the findings (14). Thus, PCA-derived DPs are highly population specific. However, the PCA is an important tool to reduce multidimensional data to lower dimensions while retaining most of the information and was therefore useful in identifying important DPs in this population.

## Conclusion

In conclusion, this study indicates contextual differences in dietary habits and their resultant association with predicted 10-y ASCVD risk. There was an inverse association between ‘mixed’ and ‘rice, pasta, meat, and fish’ DPs and 10-y ASCVD risk among Ghanaian populations in urban Ghana. This suggests that similar to other populations, diet could be an important predictor of cardiovascular health in this population. We recommend further studies to investigate underlying contextual factors related to the wider food environment such as availability, accessibility, and dietary habits and the interrelation between these, possibly through a system-wide approach, to support context-specific dietary recommendations for CVD prevention among sub-Saharan African populations.

## Supplementary Material

nxz002_Supplemental_File

## Notes

The RODAM study was supported by the European Commission under the Framework Programme (Grant Number: 278901). DB is supported by the Global Health Scholarship Programme, University Medical Center Utrecht, The Netherlands. CG is supported by NutriAct – Competence Cluster Nutrition Research Berlin-Potsdam funded by the German Federal Ministry of Education and Research (FKZ: 01EA1408A-G).

Author disclosures: DB, CG, MN, KM, EB, LS, HAO-K, SB, PA-B, FPM, JS, DEG, MBS, KS, CA, ID, and KK-G, no conflicts of interest.

Supplemental Tables 1–6 are available from the “Supplementary data” link in the online posting of the article and from the same link in the online table of contents at https://academic.oup.com/jn/.

Abbreviations used: ACC, American College of Cardiology; ASCVD, atherosclerotic cardiovascular disease; CVD, cardiovascular disease; DP, dietary pattern; GI, glycemic index; LMIC, low- and middle-income countries; PCA, principal component analysis; PCE, pooled cohort equations; PR, prevalence ratio; T2D, type 2 diabetes.
