# Supplementary material for: Dietary Patterns Are Associated with Predicted 10-Year Risk of Cardiovascular Disease Among Ghanaian Populations: the Research on Obesity and Diabetes in African Migrants (RODAM) Study
Source: J Nutr. 2019 Apr 24;149(5):755–69. doi: 10.1093/jn/nxz002 (PMC6533550; doi:10.1093/jn/nxz002)
Supplement: nxz002_Supplemental_File [file nxz002_supplemental_file.pdf]

Supplementary Data

Supplemental Table 1: Dietary patterns derived by principal component analysis and rotated factor loadings

Food groups	Dietary patterns		
	"Mixed"	"Rice, pasta, meat and fish"	"Roots, tubers and plantain"
Whole grain cereals	0.71*	-0.03	-0.08
Refined cereals	-0.05	0.02	0.57*
Sweet spreads	0.42*	0.1	0.01
Dairy products	0.49*	0.3*	-0.01
Fruits	0.31*	0.06	0.57*
Nuts and seeds	0.15	0.21	0.32*
Roots, tubers, plantain	-0.17	0	0.72*
Potatoes	0.56*	0.13	0.06
Fermented maize products	-0.2	0.06	0.67*
Vegetables	0.54*	0.14	0.05
Legumes	-0.09	0.38*	0.41*
Vegetable soups, stews, sauces	-0.01	0.05	-0.09
Rice and pasta	0.08	0.54*	-0.1
Egg	0.15	0.47*	-0.03
Red meat	0.03	0.65*	0.12
Poultry	0.51*	0.26	0.17
Processed meat	0.25	0.46*	-0.03
Fish	-0.17	0.4*	0.12
Meaty mixed dishes	-0.17	0.5*	0.17
Vegetarian mixed dishes	-0.36*	0.19	0.03
Cakes and sweets	0.24	0.52*	0.06
Coffee and tea	0.65	-0.02	-0.06
Alcoholic beverages	0.18	0.07	-0.04
Sodas and juices	0.41*	0.25	-0.01
Palm oil	-0.42*	0.02	0.32*
Olive oil	0.47*	-0.1	0.04
Other oils	0.32*	0.03	0.07
Margarine	0.39*	-0.02	0.19
Cooking fats	0.11	0.02	0.15
Condiments	0.43*	0.56*	-0.15

**Rotated factor loadings of* $\geq |0.30|$ *were considered to contribute to the pattern*

Supplementary Data

Supplementary Table 2: Demographic characteristics and risk factor profile stratified by RODAM study site[1]

Variables	Total (*n*=2976)	Europe (*n*=1393)	Urban Ghana (*n*=944)	Rural Ghana (*n*=639)
**Age, y**	51.7 ± 7.6	50.9 ± 6.9	51.8 ± 7.6	53.6 ± 8.8
**Sex (Male), %**	38.1	44.4	29.9	36.8
**Education[2], %**				
Never or elementary	40.3	25.1	48.1	61.8
Low	35.5	37.6	36.9	29.0
Intermediate	16.7	25.1	10.8	6.9
Higher vocational or University	7.5	12.1	4.2	2.3
**Systolic Blood pressure, mmHg**	134 ± 19.8	138 ± 17.6	131 ± 20.5	129 ± 21.8
**Use of antihypertensive, %**	24.2	36.9	15.1	9.9
**Serum total cholesterol, mmol/L**	5.1 ± 1.2	5.2 ± 1.1	5.4 ± 1.2	4.7 ± 1.2
**Serum HDL cholesterol, mmol/L**	1.3 ± 0.4	1.4 ± 0.3	1.3 ± 0.3	1.2 ± 0.4
**Serum LDL Cholesterol, mmol/L**	3.3 ± 1.0	3.3 ± 1.0	3.6 ± 1.0	2.9 ± 1.0
**Smoking, %**	2.8	4.5	0.8	2.0
**Diabetes, %**	11.9	14.1	12.5	6.3
**BMI, kg/m^2**	27.1 ± 5.4	29.0 ± 4.7	27.3 ± 5.2	22.5 ± 4.4
≥ 25 to <30kg/m^2	36.1	43.9	36.6	18.2
≥30 kg/m^2	27.7	37.9	27.5	5.7
**Total energy intake, kcal/d**	2478 ± 827	2610 ± 891	2238 ± 661	2544 ± 828
**Physical activity[3], METs-h/week, median (25th, 75th percentile)**	64.5 (12.0, 168)	72.0 (15.0, 189)	56.0 (6.0, 150)	92.0 (36.0, 174)
**High ASCVD risk, %**	33.6	36.5	31.0	31.1
**Length of stay in Europe, yr**		19.3 ± 9.2		

[1]*Data are percentages, mean ± SD, or median (25th, 75th percentiles). ASCVD, cardiovascular disease; BMI, body mass index; HDL, high density lipoprotein; LDL, low density lipoprotein; MET, metabolic equivalent of task*

[2]*Low education: Elementary=Primary or basic school education; Low=Lower/ Junior secondary; Intermediate= intermediate vocational schooling or intermediate/higher secondary schooling, sixth form or college; Higher vocational or tertiary=tertiary including university or polytechnic*

[3]*Physical activity was assessed using the World Health Organization (WHO) STEPwise approach to chronic disease risk factor Surveillance (STEPS) Questionnaire*

Supplementary Data

Supplemental Table 3: Intakes of food groups across the quintiles of dietary patterns' scores in Europe[1]

Food groups	Mixed dietary pattern			*P*-trend	Rice, pasta, meat and fish dietary pattern			*P*-trend	Root, tubers and plantain pattern			*P*-trend
	Q1 N=278	Q3 N=279	Q5 N=278		Q1 N=278	Q3 N=279	Q5 N=278		Q1 N=278	Q3 N=279	Q5 N=278	
Whole grain, bread, cereals, g/d	42.2±29.3	70.6±40.5	124.9±84.7	<0.001	87.1±73.6	73.6±53.5	71.8±55.8	0.001	67.1±53.7	73.5±52.2	93.4±72.4	<0.001
Refined cereals, g/d	55.0±65.9	51.1±62.4	88.0±88.1	<0.001	68.8±81.6	60.1±62.6	64.8±67.4	0.810	27.8±26.2	48.8±46.8	124±103	<0.001
Sweet spreads, g/d	0.6±1.5	1.4±2.8	4.7±7.2	<0.001	1.7±3.6	1.5±3.5	2.7±5.1	<0.001	1.0±3.0	1.8±3.8	3.2±6.2	<0.001
Dairy products, g/d	67.1±65.6	85.9±86.4	153±137	<0.001	72.1±86.5	90.2±92.9	146±116	<0.001	77.0±81.0	82.8±90.3	127±118	<0.001
Fruits, g/d	162±154	232±213	426±356	<0.001	289±322	2401±237	297±278	0.440	125±87.0	219±158	51±389	<0.001
Nuts and seeds, g/d	5.3±9.3	7.5±9.3	13.1±16.6	<0.001	6.9±10.7	8.0±12.0	11.1±14.5	<0.001	3.8±4.5	6.3±8.0	16.6±18.2	<0.001
Roots, tubers & plantain, g/d	137±111	122±106	117±117	0.007	111±97.5	120±92.6	151±132	<0.001	78.5±60.2	127±86.8	177±162	<0.001
Potatoes, g/d	28.0±36.0	40.8±37.1	76.3±65.9	<0.001	39.2±45.0	41.5±38.4	67.2±62.0	<0.001	36.4±37.8	44.3±45.7	62.1±62.9	<0.001
Fermented maize products, g/d	60.8±91.1	51.4±87.1	50.7±62.5	0.125	37.7±51.6	49.2±69.7	72.4±112.1	<0.001	31.8±32.7	46.7±50.2	84.7±129	<0.001
Vegetables, g/d	246±111	346±114	455±156	<0.001	328±148.0	348±134	377±156	<0.001	295±121	355±129	427±165	<0.001
Legumes, g/d	39.5±35.8	44.0±34.4	56.6±43.1	<0.001	30.5±25.1	41.1±31.5	63.0±46.5	<0.001	27.5±20.5	42.7±31.4	65.7±48.2	<0.001
Vegetable soups, stews, sauces, g/d	521±468	453±441	374±383	<0.001	416±430	447±435	465±433	0.682	618±482	379±403	352±372	<0.001
Rice and pasta, g/d	168±99.0	146±89.4	157±96.0	0.535	84.9±68.3	163±80.1	220±98.8	<0.001	198±93.4	141±85.8	145±99.3	<0.001
Egg, g/d	8.4±10.6	8.8±10.4	11.0±13.3	0.002	4.7±5.6	8.1±8.8	17.7±15.9	<0.001	10.9±12.8	8.7±10.7	9.2±11.0	0.165
Red meat, g/d	40.6±39.3	36.5±30.5	38.8±33.7	0.685	20.2±18.4	31.6±24.4	70.9±44.9	<0.001	40.3±36.3	33.7±28.7	42.5±38.5	0.139

Supplementary Data

Poultry, g/d	37.2±27.8	46.6±26.6	51.1±26.9	<0.001	33.8±26.6	45.5±26.4	56.2±24.8	<0.001	56.5±25.5	43.0±26.9	41.5±27.0	<0.001
Processed meat	10.7±14.4	12.6±17.1	17.5±28.6	<0.001	6.7±8.8	10.0±10.4	27.4±33.1	<0.001	14.7±22.0	9.9±16.9	13.0±16.8	0.785
Fish, g/d	53.5±34.6	55.4±32.7	60.6±36.6	0.033	40.0±30.3	57.0±30.9	70.7±36.1	<0.001	50.6±33.0	58.0±33.8	62.7±37.1	<0.001
Meaty mixed dishes, g/d	56.7±51.8	35.1±37.7	35.2±39.0	<0.001	23.3±30.6	36.1±34.3	73.6±53.6	<0.001	36.3±40.6	40.8±42.1	46.0±44.9	0.006
Vegetarian mixed dishes, g/d	21.0±23.8	10.3±14.5	12.9±17.7	<0.001	8.1±12.2	11.9±16.3	24.1±22.9	<0.001	12.7±17.0	11.9±16.7	19.2±22.3	<0.001
Cakes and sweets, g/d	19.0±17.1	22.8±25.1	28.8±30.2	<0.001	12.0±9.4	21.4±16.1	40.3±40.2	<0.001	21.2±23.8	20.3±18.3	27.8±32.1	0.001
Coffee and tea, g/d	383±370	848±556	1292±1050	<0.001	967±950	819±618	751±737	<0.001	737±631	910±768	91±857.4	<0.001
Alcoholic beverages, g/d	85.8±270.0	101±210	130±286	0.007	90.3±283	94.3±266	147±300	0.002	109±292	61.4±146.0	115±261	0.674
Sodas and juices, g/d	156±244	270±442	480±641	<0.001	201±374	259±411	477±673	<0.001	255±388	242 ±418	337±540	0.001
Palm oil, g/d	0.6±1.5	0.3±0.6	0.4±0.8	<0.001	0.4±0.7	0.3±0.6	0.4±0.9	0.179	0.2±0.4	0.3±0.6	0.7±1.6	<0.001
Olive oil, g/d	0.5±0.8	0.8±1.3	2.1±3.0	<0.001	1.9±2.8	.07±1.3	0.8±1.6	<0.001	0.4±1.0	0.8±1.5	2.0±2.7	<0.001
Other oils, g/d	4.8±8.3	8.3±12.0	14.4±20.5	<0.001	11.2±19.1	8.7±13.1	7.9±11.8	0.007	4.8±9.3	9.0±12.9	14.2±19.4	<0.001
Margarine, g/d	2.7±3.4	4.3±4.2	7.3±9.4	<0.001	5.9±8.8	4.7±5.7	4.2±4.5	0.001	3.2±3.5	4.5±4.6	7.3±9.1	<0.001
Cooking fats, g/d	0.0±0.1	0.0±0.1	0.1±0.4	<0.001	0.0±0.1	0.0±0.1	0.1±0.2	0.7242	0.0±0.1	0.0±0.1	0.1±0.4	<0.001
Condiments , g/d	86.5±54.8	102±56	116±64.8	<0.001	51.3±38.0	102±43.1	152±55.0	<0.001	118±54.6	97.9±55.4	92.6±66.0	<0.001

[1] ***Data are presented as mean ± SD***

Supplementary Data

Supplemental Table 4: Intakes of food groups across the quintiles of dietary patterns' scores in urban Ghana[1]

Food groups	Mixed dietary pattern			*P*-trend	Rice, pasta, meat and fish dietary pattern			*P*-trend	Root, tubers and plantain pattern			*P*- trend
	Q1 N=188	Q3 N=189	Q5 N=189		Q1 N=188	Q3 N=189	Q5 N=189		Q1 N=188	Q3 N=189	Q5 N=189	
Whole grain, bread, cereals, g/d	2.4±6.2	11.9±1402	49.1±65.8	<0.001	19.2±46.9	15.5±28.7	20.5±36.1	0.722	9.9±27.2	20.9±40.5	19.6±27.7	0.024
Refined cereals, g/d	105±61.7	117±85.4	139±96.4	<0.001	126±90.6	113±71.8	120±85.6	0.387	68.6±31.0	11±70.6	187±99.8	<0.001
Sweet spreads, g/d	0.1±0.3	0.1±0.6	0.5±2.0	<0.001	0.1±0.2	0.2±0.9	0.2±0.5	0.117	0.2±0.9	0.1±0.5	0.2±1.7	0.507
Dairy products, g/d	31.3±39.6	40.7±42.0	78.2±68.8	<0.001	24.0±30.8	45.7±56.1	73.6±53.9	<0.001	32.0±39.1	50.8±50.8	65.0±66.5	<0.001
Fruits, g/d	113±94.2	141±123	205±149	<0.001	135±143	149±132	180±142	<0.001	76.6±63.9	142±94.9	255±189	<0.001
Nuts and seeds, g/d	68±6.5	7.4±8.5	12.8±11.1	<0.001	4.0±5.3	8.5±9.0	13.3±10.5	<0.001	3.8±3.8	8.0±7.0	15.1±12.1	<0.001
Roots, tubers & plantain, g/d	182±98.0	195±121	213±161	0.014	193±150	197±144	205±142	0.311	133±69	182±92.0	289±210	<0.001
Potatoes, g/d	1.5±4.9	2.0±5.2	8.4±31.7	<0.001	1.5±5.3	5.5±29.0	4.0±11.7	0.048	1.4±3.8	2.7±9.4	4.4±25.7	0.001
Fermented maize products, g/d	138±105	159±175	166±158	0.027	131±141	138±120	178±152	0.001	82.5±60.8	134±84.6	268±232	<0.001
Vegetables, g/d	173±74.4	287±97.2	325±97.4	<0.001	250±115	253±103	288±103	0.001	225±104	276±108	288±101	<0.001
Legumes, g/d	66.4±47.3	57.0±40.0	62.0±42.6	0.423	33.9±27.5	62.1±41.4	87.7±45.2	<0.001	34.1±25.7	58.4±39.3	77.2±47.9	<0.001
Vegetable soups, stews, sauces, g/d	516±412	428±358	517±410	0.535	445±381	447±370	567±429	0.008	571±446	484±402	415±330	<0.001
Rice and pasta, g/d	159±82.8	168±88.5	171±85.7	0.060	90.6±66.6	176±82.8	222±71.3	<0.001	188±99.4	155±77.5	156±86.2	0.001
Egg, g/d	7.2±9.6	5.5±6.5	6.7±8.6	0.381	2.9±3.8	5.5±6.	13.4±13.0	<0.001	7.0±9.6	5.8±7.3	6.4±8.5	0.392
Red meat, g/d	49.1±46.0	31.8±31.7	43.5±42.4	0.166	13.1±13.0	32.1±24.4	86.3±42.0	<0.001	32.5±34.9	37.7±37.3	49.1±42.9	<0.001

Supplementary Data

Poultry, g/d	11.1±12.6	14.8±14.9	20.7±17.0	<0.001	6.6±7.6	13.9±13.8	23.7±18.8	<0.001	14.3±15.8	15.0±14.1	15.9±15.7	0.143
Processed meat	7.2±15.1	5.1±15.6	14.9±28.1	<0.001	1.1±3.7	5.1±10.6	23.±36.2	<0.001	8.6±20.3	5.9±16.1	10.5±25.2	0.170
Fish, g/d	106±34.1	84.0±34.0	87.9±32.7	<0.001	67.8±32.1	90.1±32.2	105±31.9	<0.001	78.7±34.3	88.6±32.1	95.6±38.1	<0.001
Meaty mixed dishes, g/d	58.0±49.5	56.2±48.0	60.9±49.1	0.898	22.9±29.7	54.0±44.3	101±38.2	<0.001	43.1±39.6	55.3±46.9	69.4±52.6	<0.001
Vegetarian mixed dishes, g/d	36.5±12.8	32.1±14.3	24.5±15.9	<0.001	31.3±16.0	31.4±15.4	27.0±13.3	0.001	33.7±14.0	31.2±14.9	25.5±15.8	<0.001
Cakes and sweets, g/d	16.1±13.6	17.6±21.6	25.6±26.7	<0.001	7.9±5.7	15.6±9.6	39.7±29.3	<0.001	13.6±13.9	18.6±20.0	24.2±23.1	<0.001
Coffee and tea, g/d	74.4±80.2	130±172	299±449	<0.001	175±323	135±194	176±235	0.776	142±261	136±228	222±359	<0.001
Alcoholic beverages, g/d	20.9±62.4	26.7±172	39.3±239	0.333	7.4±37.8	15.2±39.1	64.0±281	<0.001	31.2±223	13.6±64.6	19.1±65.5	0.544
Sodas and juices, g/d	44.6±71.3	113±259	372±505	<0.001	92.4±269	96.6±232	216.4±370	<0.001	57.9±144	174±352	196±377	<0.001
Palm oil, g/d	2.6±2.3	1.8±1.5	1.8±1.5	<0.001	1.8±1.7	2.1±1.8	2.2±1.8	0.031	1.3±0.9	2.1±1.6	2.8±2.4	<0.001
Olive oil, g/d	0.0±0.1	0±0.1	0.2±0.8	<0.001	0.1±0.4	0.0±0.3	0.0±0.4	0.555	0.0±0.1	0.1±0.6	0.1±0.5	0.003
Other oils, g/d	5.4±5.0	4.3±4.1	5.8±6.5	0.129	3.3±3.7	5.1±5.4	7.2±5.7	<0.001	4.0±4.8	4.6±3.8	6.7±7.8	<0.001
Margarine, g/d	0.7±2.0	1.8±3.2	4.1±8.8	<0.001	2.5±8.0	2.0±3.1	2.5±4.5	0.697	1.3±2.8	1.7±2.7	3.5±8.8	<0.001
Cooking fats, g/d	0.0±0.0	0.0±0.1	0.0±0.4	0.008	0.0±0.1	0.0±0.1	0.0±0.0	0.900	0.0±0.0	0.0±0.0	0.±0.3	<0.001
Condiments , g/d	50.5±34.1	60.8±29.9	67.2±36.5	<0.001	33.9±18.5	58.5±25.1	86.6±36.4	<0.001	64.4±32.8	56.8±28.6	54.5±34.6	0.002

[1]***Data are presented as mean ± SD***

Supplementary Data

Supplemental Table 5: Intakes of food groups across the quintiles of dietary patterns' scores in rural Ghana[1]

Food groups	Mixed dietary pattern			*P*- trend	Rice, pasta, meat and fish dietary pattern			*P*-trend	Root, tubers and plantain pattern			*P*- trend
	**Q1** **N=127**	**Q3** **N=128**	**Q5** **N=128**		**Q1** **N=127**	**Q3** **N=128**	**Q5** **N=128**		**Q1** **N=127**	**Q3** **N=128**	**Q5** **N=128**	
Whole grain, bread, cereals, g/d	2.6±7.3	4.5±7.6	22.4±40.3	<0.001	8.2±19.5	7.4±16.1	7.5±22.3	0.990	7.1±13.3	9.2±24.5	11.9±29.8	0.003
Refined cereals, g/d	110±80.6	119±90.0	177±134	<0.001	121±112	117±93.0	150±89.6	0.026	69.1±34.7	117±86.6	209±130	<0.001
Sweet spreads, g/d	0.0±0.1	0.1±0.3	0.6±4.1	<0.001	0.1±0.2	0.0±0.1	0.2±1.0	0.737	0.0±0.1	0.1±0.8	0.2±1.0	0.160
Dairy products, g/d	145±19.4	22.1±25.8	35.6±44.9	<0.001	12.4±16.4	19.9±27.5	38.5±46.3	<0.001	19.6±26.2	21.9±26.4	28.9±38.7	<0.001
Fruits, g/d	173±166	237±197	635±465	<0.001	264±277	295±316	439±401	<0.001	115±86.9	267±217	580±447	<0.001
Nuts and seeds, g/d	6.1±7.9	7.1±7.4	9.1±7.7	0.024	4.3±5.8	6.0±7.5	9.4±11.0	<0.001	4.3±5.0	6.1±6.8	10.7±12.3	<0.001
Roots, tubers & plantain, g/d	309±188	347±247	418±275	<0.001	382±321	334±249	395±255	0.978	219±116	329±149	611±403	<0.001
Potatoes, g/d	1.6±9.3	3.8±13.0	18.2±44.2	<0.001	5.0±15.7	5.0±17.7	9.6±26.2	0.012	3.5±11.9	5.8±21.2	13.2±40.2	0.002
Fermented maize products, g/d	195±185	209±194	213±171	0.073	184±183	211±198	250±216	0.012	119±85.2	181±119	350±284	<0.001
Vegetables, g/d	181±77.6	228±95.8	307±110	<0.001	193±98.2	237±94.8	268±113	<0.001	188±89.0	257±109	264±105	<0.001
Legumes, g/d	71.4±50.2	65.7±51.6	80.4±57.1	0.121	34.8±32.2	68.9±46.7	103±57.7	<0.001	36.6±29.6	69.4±48.5	102±52.6	<0.001
Vegetable soups, stews, sauces, g/d	446±326	399±284	377±201	0.036	425±344	446±325	411±227	0.434	529±409	407±285	369±186	<0.001
Rice and pasta, g/d	133±76.6	115±87.9	144±104	0.287	55.2±48.2	128±72.0	197±91.1	<0.001	130±80.5	111±83.9	136±96.2	0.315
Egg, g/d	5.6±9.4	5.1±.8.8	7.2±10.4	0.014	1.9±2.9	3.6±6.1	12.5±14.2	<0.001	4.8±8.5	5.2±9.3	8.2±11.6	0.001
Red meat, g/d	38.9±40.0	29.6±29.1	39.7±34.6	0.643	14.0±12.4	26.2±23.1	68.0±43.0	<0.001	25.7±26.2	30.1±31.3	42.5±37.7	<0.001
Poultry, g/d	10.3±12.8	12.3±14.7	21.0±20.0	<0.001	9.4±13.9	11.8±12.6	25.6±21.4	<0.001	11.6±12.5	14.3±16.1	15.3±17.0	0.031

Supplementary Data

Processed meat	1.3±7.2	3.0±10.4	4.4±13.7	<0.001	0.6±2.7	0.8±3.1	6.9±17.5	<0.001	2.2±7.9	3.0±13.7	2.6±6.0	0.399
Fish, g/d	65.8±36.5	60.7±27.3	62.7±34.9	0.509	43.3±27.7	59.2±29.9	82.2±34.5	<0.001	54.5±26.3	65.6±36.0	60.8±34.8	0.070
Meaty mixed dishes, g/d	58.7±47.6	52.0±46.8	52.7±44.9	0.074	25.1±30.2	50.7±45.0	85.0±45.9	<0.001	38.2±40.5	50.5±44.4	62.5±48.4	<0.001
Vegetarian mixed dishes, g/d	31.9±17.0	24.6±17.5	21.9±18.2	<0.001	23.2±18.3	28.1±17.4	24.6±18.1	0.656	28.7±16.0	28.0±18.2	20.9±18.0	<0.001
Cakes and sweets, g/d	11.1±15.0	13.3±14.9	18.2±21.7	<0.001	6.2±6.6	10.0±9.9	26.2±23.3	<0.001	12.0±16.7	12.3±13.0	13.0±19.0	0.003
Coffee and tea, g/d	62.8±109	74.8±107	263±589	<0.001	126±389	110±271	164±324	0.243	97.7±176	126±369	236±542	<0.001
Alcoholic beverages, g/d	19.5±62.1	31.2±91.7	50.9±302	0.272	9.8±24.1	17.0±54.6	42.1±109	0.133	52.1±306	20.2±65.0	21.3±52.1	0.208
Sodas and juices, g/d	29.8±69.5	61.4±164	162±6450	<0.001	56.6±185	63.5±226	123±614	0.001	51.8±170	135.1±618	50.9±148	0.950
Palm oil, g/d	5.2±3.3	2.4±1.7	2.2±2.2	<0.001	2.7±2.5	3.3±2.7	2.9±3.1	0.167	2.0±1.5	3.1±2.3	3.6±3.4	<0.001
Olive oil, g/d	0.0±0.1	0.0±0.0	0.1±0.3	<0.001	0.0±0.2	0.0±0.2	0.0±0.2	0.343	0.0±0.0	0.0±0.2	0.0±0.3	0.258
Other oils, g/d	3.0±3.9	2.9±2.5	3.3±4.2	0.518	2.3±3.2	2.9±2.7	4.7±6.0	<0.001	2.7±2.9	2.9±4.4	3.6±5.1	0.041
Margarine, g/d	0.5±1.6	1.5±2.5	3.0±5.6	<0.001	1.4±3.6	1.5±2.7	1.8±3.1	0.822	1.5±2.8	1.5±3.5	2.1±4.9	0.007
Cooking fats, g/d	0.0±0.1	0.1±0.2	0.3±0.8	<0.001	0.1±0.4	0.1±0.4	0.1±0.4	0.821	0.0±0.1	0.1±0.2	0.2±0.7	<0.001
Condiments , g/d	23.8±29.1	46.3±35.5	66.7±52.2	<0.001	18.7±17.6	34.6±27.4	88.8±50.5	<0.001	39.0±29.9	43.5±42.9	52.2±43.8	0.001

[1]***Data are presented as mean ± SD***

Supplementary Data

Supplemental Table 6: Multiple-adjusted associations of dietary patterns with cardiovascular disease risk factors by study site, the RODAM study[1]

Models	Serum total cholesterol		Serum LDL cholesterol		Serum HDL cholesterol		Systolic blood pressure		Type 2 diabetes	
	β[2] per 1 score-SD increase (95% CI)	*P*-value	β[2] per 1 score-SD increase (95% CI)	*P*-value	β[2] per 1 score-SD increase (95% CI)	*P*-value	β[2] per 1 score-SD increase (95% CI)	*P*-value	PR[3] per 1 score-SD increase (95% CI)	*P*-value
**Europe**										
Mixed dietary patterns										
**Crude**	-0.03 [-0.09, 0.03]	0.289	-0.04 [-0.09, 0.02]	0.159	0.02 [-0.00, 0.04]	0.059	-0.02 [-0.94, 0.91]	0.973	0.99 [0.87, 1.13]	0.913
**Model 1**	-0.04 [-0.10, 0.02]	0.163	-0.05 [-0.10, 0.01]	0.096	0.02 [-0.00, 0.03]	0.076	-0.07 [-0.99, 0.85]	0.881	0.96 [0.84, 1.09]	0.516
**Model 2**	-0.06 [-0.12, 0.01]	0.073	-0.05 [-0.11, 0.00]	0.069	0.01 [-0.01, 0.03]	0.301	-0.09 [-0.96, 0.79]	0.859	1.02 [0.89, 1.17]	0.753
Rice, pasta, meat and fish										
**Crude**	0.05 [-0.01, 0.11]	0.075	0.02 [-0.03, 0.08]	0.928	0.02 [0.00, 0.04]	0.025	-0.32 [-1.24, 0.61]	0.501	0.83 [0.72, 0.96]	0.011
**Model 1**	0.06 [-0.01, 0.11]	0.072	0.02 [-0.03, 0.07]	0.433	0.03 [0.02, 0.04]	0.004	-0.23 [-1.15, 0.69]	0.621	0.87 [0.76, 1.00]	0.053
**Model 2**	0.05 [-0.01, 0.11]	0.128	0.02 [-0.04, 0.08]	0.500	0.02 [0.00, 0.04]	0.018	-0.27 [-1.25, 0.72]	0.594	0.90 [0.78, 1.05]	0.176
Roots, tubers and plantain										
**Crude**	-0.01 [-0.03, 0.01]	0.264	-0.01 [-0.06, 0.04]]	0.773	-0.00 [-0.02, 0.02]	0.927	0.78 [-0.14, 1.70]	0.098	1.05 [0.92, 1.19]	0.486
**Model 1**	-0.03 [-0.09, 0.03]	0.103	-0.02 [-0.07, 0.03]	0.484	-0.01 [-0.02, 0.02]	0.804	0.51 [-0.41, 1.43]	0.275	1.01 [0.89, 1.14]	0.903
**Model 2**	-0.04 [-0.10, 0.03]	0.253	--0.02 [-0.07, 0.04]	0.483	-0.01 [-0.03, 0.01]	0.264	0.69 [-0.25, 1.62]	0.150	1.06 [0.93, 1.21]	0.406
**Urban Ghana**										

Supplementary Data

Mixed dietary patterns										
**Crude**	-0.04[-0.10, 0.03]	0.311	-0.02 [-0.08, 0.05]	0.557	-0.00 [-0.02, 0.02]	0.914	-1.05 [[-2.37, 0.26]	0.116	0.79 [0.66, 0.96]	0.018
**Model 1**	-0.03 [-0.10, 0.04]	0.408	-0.02 [-0.08, 0.05]	0.597	-0.00 [-0.02, 0.02]	0.926	-0.94 [-2.22, 0.34]	0.149	0.82 [0.66, 1.01]	0.066
**Model 2**	-0.05 [-0.13, 0.03]	0.201	-0.04 [-0.10, 0.03]	0.311	0.01 [-0.02, 0.03]	0.551	-1.02 [-2.38, 0.35]	0.146	0.83 [0.67, 1.03]	0.088
Rice, pasta, meat and fish										
**Crude**	0.02 [-0.06, 0.09]	0.631	0.02 [-0.04, 0.09]	0.481	-0.01 [-0.03, 0.01]	0.429	-1.92 [-3.22, -0.61]	0.004	0.79 [0.66, 0.95]	0.012
**Model 1**	0.05 [-0.02, 0.13]	0.175	0.04 [-0.02, 0.11]	0.193	0.01 [-0.02, 0.04]	0.759	-1.34 [-2.67, -0.01]	0.049	0.83 [0.69, 1.01]	0.053
**Model 2**	0.05 [-0.04, 0.14]	0.309	0.04 [-0.04, 0.11]	0.389	0.01 [-0.02, 0.03]	0.723	-1.32 [-2.91, 0.28]	0.105	0.91 [0.70, 1.18]	0.476
Roots, tubers and plantain										
**Crude**	-0.04 [-0.11, 0.04]	0.310	-0.02 [-0.08, 0.05]	0.631	-0.00 [-0.02, 0.02]	0.901	-0.46 [-0.18, 0.86]	0.497	0.89 [0.73, 1.09]	0.267
**Model 1**	-0.03 [-0.10, 0.05]	0.444	-0.01 [-0.07, 0.05]	0.753	-0.00 [-0.02, 0.02]	0.951	-0.27 [-0.16, 1.02]	0.685	0.92 [0.75, 1.12]	0.916
**Model 2**	-0.02 [-0.10, 0.07]	0.727	0.01 [-0.06, 0.08]	0.797	-0.00 [-0.03, 0.02]	0.782	0.76 [-0.74, 2.27]	0.318	1.00 [0.80, 1.26]	0.970
**Rural Ghana**										
Mixed dietary patterns										
**Crude**	0.02 [-0.07, 0.12]	0.639	0.01 [-0.07, 0.09]	0.760	0.02 [-0.02, 0.05]	0.305	0.64 [-1.05, 2.33]	0.458	0.96 [0.69, 1.35]	0.964
**Model 1**	0.02 [-0.07, 0.10]	0.729	0.01 [-0.07, 0.08]	0.846	0.02 [-0.02, 0.05]	0.325	0.65 [-1.00, 2.31]	0.439	0.93 [0.65, 1.32]	0.870
**Model 2**	0.013 [-0.08, 0.11]	0.793	0.02 [-0.06, 0.10]	0.695	0.00 [-0.03, 0.04]	0.850	0.08 [-1.71, 1.88]	0.927	1.04 [0.73, 1.50]	0.815
Rice, pasta, meat and fish										

Supplementary Data

**Crude**	0.08 [-0.01, 0.18]	0.082	0.05 [-0.03, 0.13]	0.224	0.03 [0.00, 0.06]	0.052	-1.04 [-2.73, 0.65]	0.228	0.66 [0.45, 0.98]	0.041
**Model 1**	0.11 [0.02, 0.20]	0.020	0.08 [-0.00, 0.15]	0.056	0.02 [-0.01, 0.05]	0.144	-0.05 [-1.73, 1.64]	0.957	0.73 [0.50, 1.05]	0.722
**Model 2**	0.10 [0.01, 0.20]	0.041	0.08 [-0.01, 0.16]	0.070	0.02 [-0.01, 0.05]	0.235	-1.23 [-2.77, 0.31]	0.190	0.74 [0.49, 1.13]	0.162
Roots, tubers and plantain										
**Crude**	-0.03 [-0.12, 0.07]	0.594	-0.05 [-0.13, 0.03]	0.244	0.03 [-0.00, 0.06]]	0.057	0.02 [-1.67, 1.71]	0.981	0.90 [0.66, 1.22]	0.487
**Model 1**	-0.02 [-0.11, 0.07]	0.619	-0.04 [-0.11, 0.04]	0.286	0.02 [-0.01, 0.06]	0.112	0.57 [-1.09, 2.23]	0.502	0.89 [0.65, 1.22]	0.476
**Model 2**	0.10 [-0.12, 0.15]	0.828	0.01 [-0.10, 0.12]	0.839	-0.12 [-0.06, 0.03	0.2614	-0.24 [-2.72, 2.24]	0.849	1.40 [0.91, 2.15]	0.122

[1]*Model 1: Age (40-50 y, 50-60 y, 60-70 y), Sex + Education (categorical); Model 2: Model 1+ Total energy intake, kcal/d (quintiles), Physical activity, METs-h/wk (quintiles), Body mass index (kg/m²). HDL, high density lipoprotein; LDL, low density lipoprotein; PR, prevalence ratio.*

[2]*Beta-coefficients (β), 95% CIs and p-values were calculated by linear regression.*

[3]*Prevalence ratios, 95% confidence intervals (CIs) and p-values were calculated by Poisson regression with robust variance.*
